# Contact mechanics of highly porous oxide nanoparticle agglomerates

**DOI:** 10.1007/s11051-016-3500-4

**Published:** 2016-07-18

**Authors:** Andrea Fabre, Samir Salameh, Lucio Colombi Ciacchi, Michiel T. Kreutzer, J. Ruud van Ommen

**Affiliations:** Department of Chemical Engineering, Delft University of Technology, Julianalaan 136, 2628 BL Delft, The Netherlands; Hybrid Materials Interfaces University of Bremen, Bremen, Germany

**Keywords:** Young’s modulus, Porous agglomerates, Atomic force microscopy, Oxide particles, Nanoparticles, Particle characterization, Instrumentation

## Abstract

Efficient nanopowder processing requires knowledge of the powder’s mechanical properties. Due to the large surface area to volume ratio, nanoparticles experience relatively strong attractive interactions, leading to the formation of micron-size porous structures called agglomerates. Significant effort has been directed towards the development of models and experimental procedures to estimate the elasticity of porous objects such as nanoparticle agglomerates; however, none of the existing models has been validated for solid fractions below 0.1. Here, we measure the elasticity of titania (TiO$_2$, 22 nm), alumina (Al$_2$O$_3$, 8 nm), and silica (SiO$_2$, 16 nm) nanopowder agglomerates by Atomic Force Microscopy, using a 3.75 $\upmu$m glass colloid for the stress–strain experiments. Three sample preparations with varying degree of powder manipulation are assessed. The measured Young’s moduli are in the same order of magnitude as those predicted by the model of Kendall et al., thus validating it for the estimation of the Young’s modulus of structures with porosity above 90 %.

**Electronic supplementary material:**

The online version of this article (doi:10.1007/s11051-016-3500-4) contains supplementary material, which is available to authorized users.

## Introduction

The mechanical properties of nanopowders are critical for the optimization of their processing (Iwadate and Horio 1998; Bika et al. 2001; Rong et al. 2004). These properties are crucial in gas-phase processes involving agglomerate collision such as in flame aerosol reactors (Kruis et al. 1998; Madler et al. 2006), lung nanoparticle uptake studies (Limbach et al. 2005), and nanopowder fluidization (Matsuda et al. 2004). The processing method of fluidization, where the powder is suspended in a gas stream moving upwards, is known to enhance fluid to solid contact by powder dispersion (Jung and Gidaspow 2002; van Ommen et al. 2012; Dadkhah et al. 2012; Quevedo et al. 2010; Shabanian et al. 2012), which is beneficial for heat and mass transfer, and widely used in gas–solid reaction, granulation, and particle coating, drying, and mixing, among many other applications. Nonetheless, nanoparticles (NP) fluidize as clusters called agglomerates (Parveen et al. 2013; Zhou and Li 1999; Khadilkar et al. 2014; Liang et al. 2009), making the dynamics within the fluidized bed complex and challenging to study, mainly due to the lack of accurate characterization of the agglomerates (Salameh et al. 2012).

Nanopowders agglomerate in a stepwise fashion (Yao et al. 2002). During synthesis at high temperatures, primary particles form chemical bonds creating chain-like structures called aggregates, reaching sizes of 100s of nm. These aggregates then cluster together by physical interactions forming simple agglomerates with sizes of a few 10s of $\mu$m, mainly during powder storage. Finally, the simple agglomerates assemble into complex agglomerates, which can reach sizes of 100s of $\upmu$m. As a hierarchical process, each level has structures with particular features such as fractal dimension (de Martin et al. 2014; Hu et al. 2012). This research focuses on the agglomerate properties since these are the structures readily available from stored nanopowder and found during nanopowder processing in the gas phase.

Agglomeration arises from the relatively strong attractive interactions among NPs, which include capillary, solvation, van der Waals, and electrostatic forces (Laube et al. 2015; van Ommen et al. 2012; Castellanos 2005; Hakim et al. 2005; Seville et al. 2000; van Ommen 2012; Quevedo and Pfeffer 2010; Yan et al. 2010). Electrostatic forces diminish in the presence of humidity. In earlier studies, it was shown that physisorbed water molecules situate between the nanoparticles creating an attractive interaction described by a combination of capillary and solvation forces, which can surpass the van der Waals contribution (Salameh et al. 2012; Laube et al. 2015). During nanopowder processing, attractive forces are challenged by external separation forces such as collision (Matsuda et al. 2004). In order to estimate the magnitude of the forces acting on the agglomerates, and thus their morphological stability at the given conditions, their Young’s modulus must be known. The high porosity (>90 %) and rather large size of these structures ($$\approx$$$$100$$$$\upmu$$m) make them extremely fragile. Therefore, stress measurements to study their mechanical properties are particularly challenging.

Because of their large void fraction, nanoparticle agglomerates are expected to have a relatively low Young’s modulus. Due to their fragile nature, any type of manipulation can easily compromise the morphological integrity of the agglomerates and reproducibility of the results. Thus, all techniques considered for the study of agglomerates have to be evaluated for the degree of morphological modification and data reproducibility. Additionally, the number of available techniques to study structures in the micron-size scale with nanoscale resolution is very limited. A quite challenging property to measure is elasticity, mainly due to the structural and technical limitations just mentioned. There have been novel techniques to measure elasticity of soft microscopic objects such as microcapillaries, relying on pressure-induced deformation of microscopic deformable particles in a dilute suspension (Wyss et al. 2010), the ultrasonic pulse-echo method by measuring the velocity of ultrasonic waves in materials along a known crystal direction for isotropic, millimeter thick samples (Yoshimura et al. 2007; Schwarz et al. 1997), or the compression and indentation techniques such as atomic force microscopy (AFM). Methods that require the samples to be in the liquid phase, specifically oriented, or placed at a set location will considerably affect the original structure of the nanoparticle cluster and hinder result reproducibility.

Agglomerate elasticity has been measured before; however, those agglomerates had a significantly higher solid fraction, well-defined geometry, and/or customized formation process than those of interest in this research. In 1987, Kendall et al. (1987) measured the elasticity of ceramic NP clusters to study the effect of solid fraction, developing a model to estimate the effective Young’s modulus in terms of the volume packing, and particle interface energy, size, and modulus. Nonetheless, the experiments were limited to structures with porosity below 70 % (Kendall et al. 1987). Later on, in 1992, Kendall focused on the elasticity of spray-dried spherical agglomerates of uniformly packed 210-nm zirconia particles (Kendall and Weihs 1992), modeling the steps towards agglomerate fracture and describing the use of a nanoindenter to study agglomerate deformation, again, facing the porosity limitation. In 2001, Bika et al. (2001) presented a summary of studies done on the mechanical properties of wet and dry agglomerates, highlighting their morphological frailty, and the lack of proper measuring techniques and realistic theoretical models to obtain accurate values of the agglomerate mechanical properties. Nonetheless, all the data gathered from the literature and reviewed by Bika et al. is for agglomerates with porosity bellow 75 %.

The elasticity, represented as the Young’s modulus, of porous materials can be predicted from theoretical models found in the literature (Yoshimura et al. 2007). These models consider the agglomerate volume fraction and primary particle Young’s modulus as critical variables to determine the agglomerate Young’s modulus. However, the models of Hasselman (1962), Wang (1984), Martin and Haynes (1971), and Phani and Niyogi (1987) have fitting parameters that rely on elasticity experimental data, and thus not really predicting the value. The models of Yoshimura et al. (2007) and Yoshimura et al. (2007) require previous knowledge of the shear and bulk modulus of the porous structure, and Poisson’s ratio of the NP, which leads to a straightforward calculation of the elasticity. Nonetheless, these values are unknown for nanoparticle agglomerates. Kendall et al. (1987) developed a model with a simple expression that uses the agglomerate solid fraction and NP Young’s modulus, work of adhesion, and diameter to estimate the effective elasticity of the porous agglomerate, which can be obtained from commercial suppliers or literature. However, to the best of our knowledge, none of these models has been experimentally validated for structures with porosity above 90 % such as those seen in nanopowders.

A well-established technique to study the elasticity of soft matter is the AFM, which works by forcing an interaction between a probe and the sample. The versatility of the technique allows for the visualization of topographic characteristics to an atomic level, the quantification of interacting forces between nanosized objects, metal deposition on electroconductive substrates, and the measurement of mechanical properties of soft materials (Pimpang et al. 2013; Vakarelski and Higashitani 2006; Barcons et al. 2012; Sigdel et al. 2013; Salameh et al. 2014; Stiles et al. 2008; Farshchi-Tabrizi et al. 2006; Li and Chen 2014; Salameh et al. 2012; Rong et al. 2004; Webber et al. 2008; Tanabe and Tatsuma 2012). This includes fragile micron/nanosized systems such as muscle cells (Engler et al. 2004) and thin gels (Engler et al. 2004) among many other applications (Picas et al. 2012; Xu et al. 2007; Lin et al. 2007; Zheng and Ya-Pu 2004; Rong et al. 2004; Landolsi et al. 2013; Fotiadis et al. 2002; Rico et al. 2005; Dimitriadis et al. 2002; Salameh et al. 2012). In earlier studies, the AFM equipped with a glass colloid attached to the cantilever was used to measure the Young’s modulus of highly porous NP films (Schopf et al. 2013; Butt et al. 2005). To neglect extra phenomena such as adhesion forces and plasticity, only the approach part of the force curved was fitted to the Hertz model for elasticity estimations. However, these films differ from the fluidized agglomerates on the mechanism of formation, homogeneity, and stability, with porosity still bellow that of the complex nanoparticle agglomerates. This method is widely accepted for materials in the kPa–MPa range such as biological samples (Vinckier and Semenza 1998; Roduit C 2009; Radmacher et al. 1996).

The objective of this work is to present an experimental method to measure the elasticity of nanopowder agglomerates, which typically have a porosity above 90 %. The results are used to validate the applicability of elasticity models for highly porous structures. Three sample preparation approaches are compared to verify the conservation of the structure, and measurement accuracy and reproducibility. To preserve the original morphology of the agglomerate, the technique requiring the least manipulation during sample preparation is used to investigate hydrophilic titania (TiO$_2$—P25), alumina (Al$_2$O$_3$—Alu C), and silica (SiO$_2$—A130) nanopowders. The experimental results are compared to theoretical models from the literature, and the Kendall et al. (1987) method was found to give a descent estimation.

## Experimental section

### Powder characterization

**Fig. 1:**
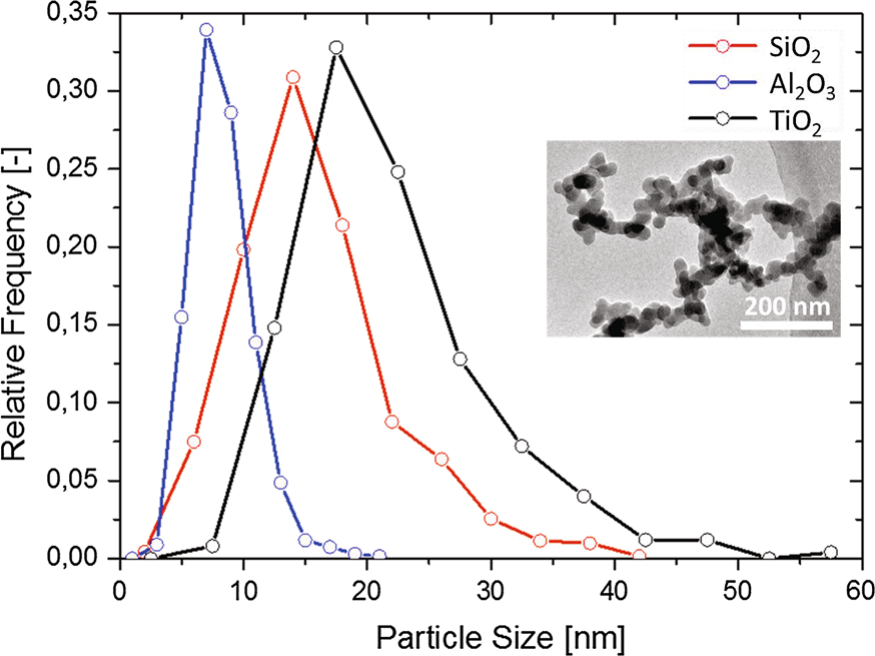
Size distribution of nanoparticles measured from TEM images using ImageJ. 250, 678, and 706 particles were counted for TiO$_2$, Al$_2$O$_3$, and SiO$_2$, respectively. The *inset* is a TEM image of Al$_2$O$_3$ showing nanoparticles connected by solid necks

The nanopowders used in this study are Aeroxide P25 (TiO$_2$), Aeroxide Alu C (Al$_2$O$_3$), and Aerosil A130 (SiO$_2$), obtained from Evonik with the specifications given in Table 1. To verify the powder characteristics, the primary particle size was determined from TEM images by manually counting 250, 678, and 706 particles for TiO$_2$, Al$_2$O$_3$, and SiO$_2$, respectively, using the open source image processing software ImageJ. The mean values obtained are $22\pm 8$, $16\pm 6$, and $8\pm 2$ nm for TiO$_2$, SiO$_2$, and Al$_2$O$_3$, respectively (Fig. 1), where the ± values are the standard deviation of each dataset. These values agree with those specified by the supplier (Table 1), with the exception of Al$_2$O$_3$, which showed a significantly lower mean size. The discrepancy could arise from the subjective particle selection during image analysis by measuring only those shades that clearly seem to be individual particles, as most of them are connected by solid necks (Fig. 1 inset). Also, the inconsistency could come from the use of different measuring techniques since the average size given from production is determined by the gas adsorption–desorption method, which could deviate from that obtained from the TEM image analysis.

**Table 1:** Properties of the nanopowders as provided by the manufacturer and obtained from TEM image analysis

powder	$\rho _p$ (kg/m$^3$)	$d_p$ (nm)	$d_{p{\mathrm{(TEM})}}$ (nm)
TiO$_2$ P25	4000	21	22 ± 8
Al$_2$O$_3$ AluC	3800	13	16 ± 6
SiO$_2$ A130	2200	16	8 ± 2

### Sample preparation

Three sample preparation methods were tested, referred to as pressed on glass, double-sided tape, and rough substrate. For the powder pressed on glass, a small amount of the nanopowder is placed on a glass microscope slide over an area of about $1cm^2$, and pressed using a thick piece of flat glass until leaving a homogeneous layer of powder looking uniform to the naked eye. The force exerted over the film is estimated using a scale reaching $12 \pm 1$ N, which results in a pressure of $9 \pm 0.5$ kPa. The double-sided tape method involves the spreading of powder over a transparent double-sided tape (Scotch) attached to a glass slide. Then, the sample is gently shaken to remove any loose powder without blowing or touching, to prevent morphological changes. Similarly, the rough surface preparation starts with the spreading of powder on the rough side of a microscope slide, with a final gentle shake to remove the excess powder. These spreading and shaking steps are repeated a few times to ensure a thick enough powder layer for AFM measurements. Due to the extent of manipulation, the pressed on glass method deliberately modifies the structure of the powder, while the sample on the rough substrate is expected to have an almost unchanged morphology. Comparably, the double-sided tape technique is believed to preserve the original structure of the agglomerates. However, the effect of the glue on the mechanical properties was questionable, and thus evaluated.

All three samples were characterized by SEM imaging. A SEM (Jeol JSM-6010 LA) was used to evaluate the general morphology of the nanopowder film on the smooth glass, rough surface, and double-sided tape. To assess the glue–powder integration, images of the tilted double-sided tape sample were taken and analyzed. The samples were slightly blown to prevent nanopowder contamination of the sample chamber. Additionally, for clear SEM imaging, the samples were coated with gold using the Auto Sputter Coater (JEOL JFC-1300).

### Elasticity measurements

**Fig. 2:**
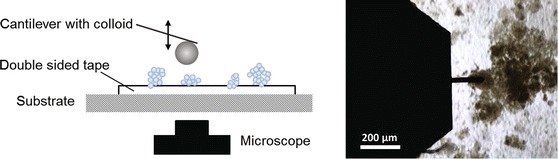
Schematic representation of the experimental setup for the double-sided tape sample preparation method. The nanopowder is attached to a glass microscope slide (substrate) using transparent double-sided tape. The 3.5-$\upmu$m colloid attached to the cantilever is aligned to the desired position on the sample with the help of a microscope located directly below the sample before each elasticity measurement. The *image* on the *right* is of Al$_2$O$_3$ on double-sided tape, taken by the AFM microscope

The stress–strain measurements were done in a Nanowizard 3 AFM from JPK. The experiments were performed using a probe with a glass colloid of 3.5 $$\upmu$$m in diameter bought from sQube (CP-FM-SiO-B) (see Fig. S1, Supporting Information). This colloid size is large enough to prevent local indentation through the primary particles, and apply pressure on an area encompassing nanoparticles agglomerates. The spring constants of 2.6, 3.5, and 3.9 N/m for Al$$_2$$O$$_3$$, SiO$$_2$$, and TiO$$_2$$ on double-sided tape, respectively, and 3.8 and 4.4 N/m for TiO$$_2$$ on a rough substrate and pressed on glass, respectively, were determined using the thermal noise method (Hutter and Bechhoefer 1993; Burnham et al. 2003). Single force curves were recorded on $$8 \times 8$$ grids in an area of $$10 \times 10$$$$\upmu$$m to average local differences. The applied force was 150 *nN* with a cantilever approach/retraction speed of 2 $$\upmu$$m/s. To avoid glue–colloid contact, the agglomerates were located before each stress–strain experiment by a microscope positioned right below the sample (Fig. 2).

The Young’s modulus was calculated using the JPKSPM Data Processing software by fitting the Hertz model (Hertz 1881) to the approach curves. First, the baseline was subtracted from the curves to set the point of cantilever–sample contact at zero and have displacement equal to indentation. Then, the x offset (contact point) was adjusted and the height for cantilever bending, corrected previous to the Young’s modulus estimation using the embedded “determine elasticity from indentation” software function. Retraction curves were not considered for elasticity measurements of the agglomerates; hence, only the approach curves are presented and used for the estimation of the Young’s modulus. Other contact mechanics models such as Johnson–Kendall–Roberts (JKP) (Johnson et al. 1971), Derjaguin–Muller–Toporov (DMT) (Derjaguin et al. 1975), and Maugis–Dugdale (MD) (Maugis 1992), which account for adhesion forces (Lin et al. 2007; Landolsi et al. 2013), were also considered.

## Results and discussion

### Sample characterization

From the TEM pictures, it is evident that nanoparticles are found in clusters. These structures are very porous and expected to be susceptible to changes by external disturbances. Therefore, any powder manipulation and processing will dramatically modify their original morphology. Insufficient analysis and understanding of the handling effect can lead to erroneous conclusions regarding the nature of the nanoparticle clusters.

**Fig. 3:**
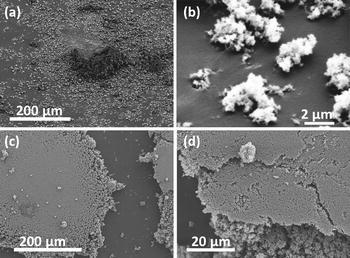
SEM pictures of TiO$_2$ nanopowder samples; **a**, **b** spread on double-sided tape; **c**, **d** pressed on glass. The porosity, distribution, and morphology of the powder clusters are noticeably different between the double-sided tape and pressed on glass samples. Images (**a**, **b**) show the agglomerates attached to the double-sided tape. The pressed powder cluster shows a very uniform flat surface with a few loose agglomerates on top (**c**), and an edge that resembles the structure of the spread powder (**d**)

Sample preparation was thoroughly evaluated to prevent false conclusions due to the fragility of the agglomerates. The soft spreading and gentle shake for the rough surface and double-sided tape sample preparation methods show fluffy structures, as expected from unprocessed nanopowder (Fig. 3a, b). On the other hand, the powder pressed on glass shows a flat and dense surface arising from the pressing step (Fig. 3c). Nonetheless, the pressed film seems to keep a highly porous morphology underneath the flat surface (Fig. 3d). The SEM images showed a morphology similar to naturally formed complex agglomerates for the rough surface and double-sided tape samples, while there was considerable modification on the pressed on glass nanopowder film.

Besides the preparation method, the sample substrate could also affect the AFM measurements. From SEM images, it was seen that the powder film thickness is considerably larger than the colloid indentation depth (Fig. 3), and since the elasticity of the solid substrates is known to be orders of magnitude higher than that of the porous film, the substrates should not have an effect on the measurements. However, the possibility of glue penetration by capillary into the highly porous structures led to extra evaluation of samples placed on the double-sided tape. These samples were assessed by tilted SEM imaging, where the glue was found to immerse less than 0.5 $\upmu$m of the attached agglomerates (Fig. 3b). Thus, the glue, as well as the solid substrates, is expected to have negligible to no influence on the AFM measurements, leaving any measurement discrepancy to the preparation method itself.

### Force curve analysis

**Fig. 4:**
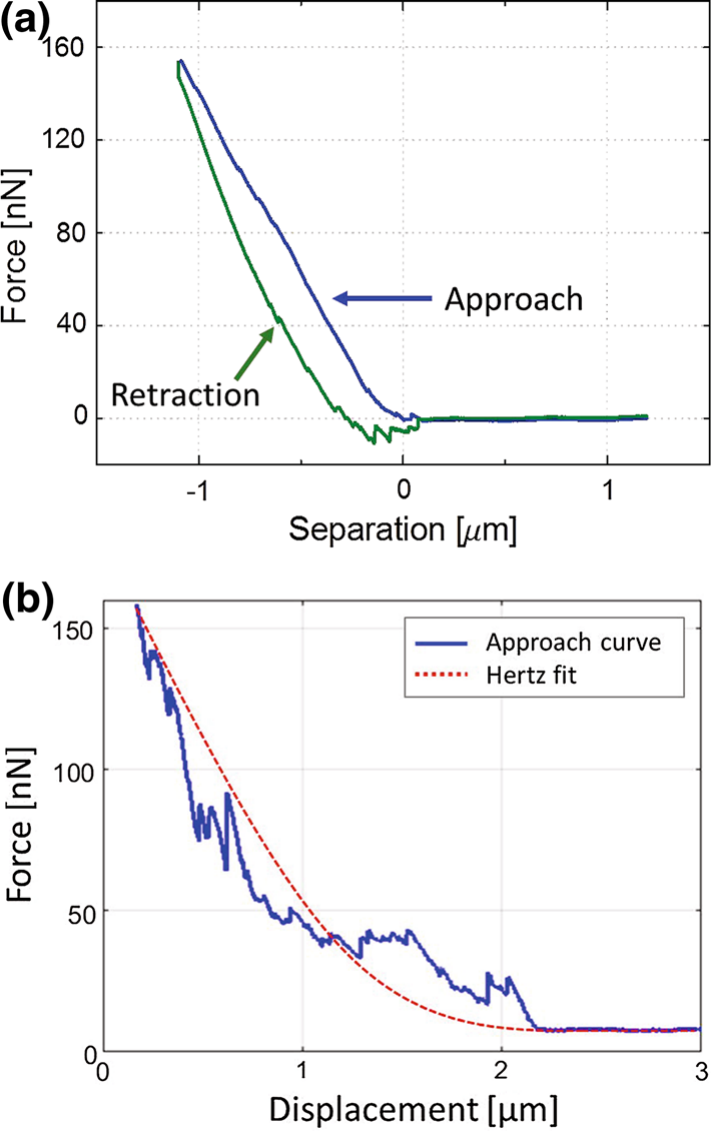
**a** Full force curve of an alumina (Al$_2$O$_3$) sample measured by AFM. The approach part of the curve is shown in *blue* and the retraction part, in *green*. The *horizontal axis* is the separation between the colloid and the sample. The hysteresis between the approach and retraction curves, in addition to the multiple peaks on the retraction curve, suggests elastic deformation of the sample. **b** Force versus displacement curve showing particle rearrangement. Example of a plot not included in the analysis. (Color figure online)

For an ideal elastic sample, the slope of approach and retraction part does not differ. However, in the case of the highly porous agglomerates, there is a large hysteresis between approach and retraction (Fig. 4a). To investigate the elasticity of porous samples by AFM, the approach part of the force curve should be analyzed (Butt et al. 2005). This is due to the complexity of the retraction curve, which includes other phenomena such as strong short-range adhesion forces between the colloid and the agglomerate that lead to deformation of the agglomerate while the cantilever retracts. Moreover, a certain amount of approach curves (<33 %) show plastic deformations and an inaccurate fit of the Hertz model (Fig. 4b),and hence were eliminated from data analysis (see Figs. S2 and S3, Supporting Information).

**Fig. 5:**
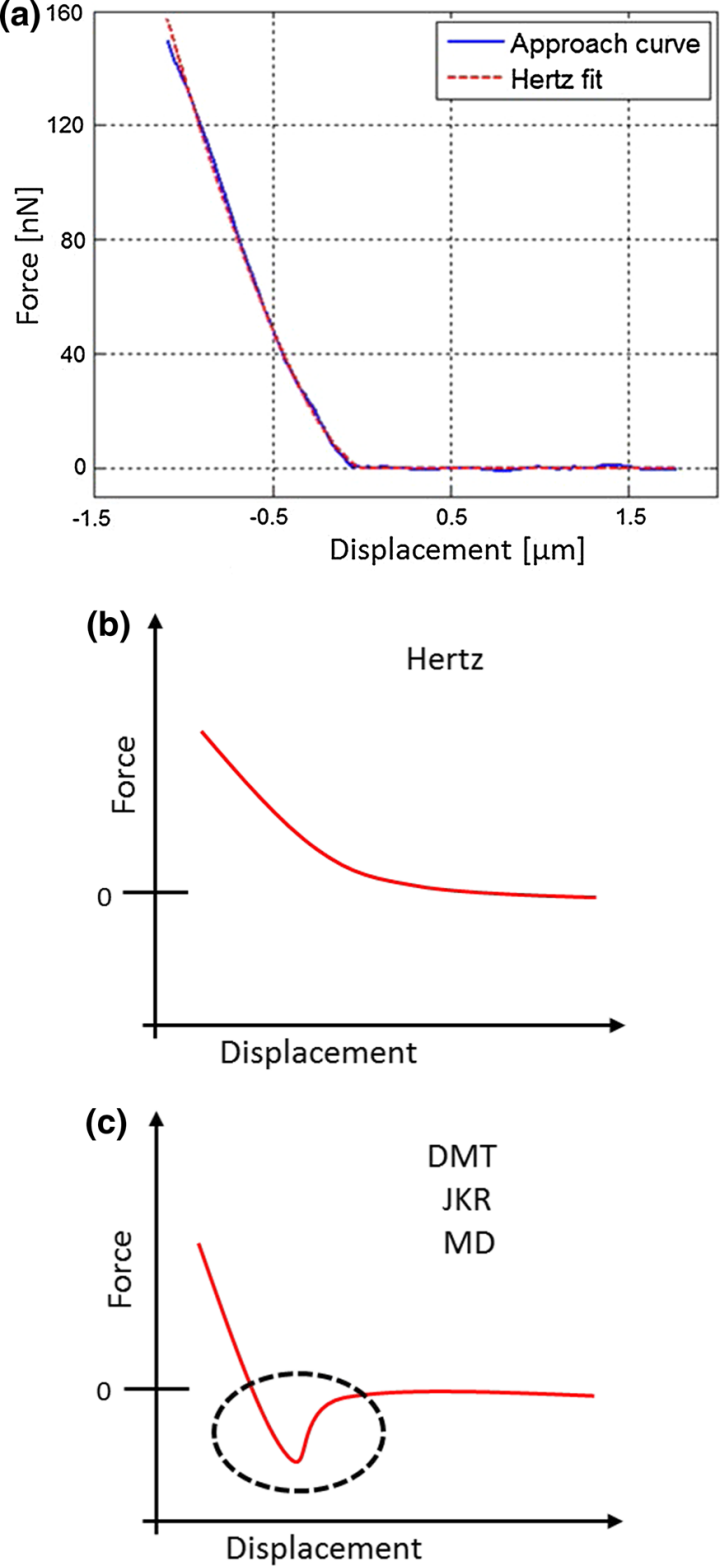
Hertz fit to the approach part of an experimental force versus displacement curve (**a**). Hertz contact model curve in a force versus displacement plot (**b**). General force versus displacement curve for the DMT, JKR, and MD models, which account for the effect of adhesion (**c**)

The approach part of the force curves obtained from the AFM measurements were fitted using the Hertz model (Hertz 1881) to calculate the Young’s modulus of each sample (Fig. 5a). A total of five samples were analyzed, consisting of Al$_2$O$_3$, SiO$_2$, and TiO$_2$ on double-sided tape, TiO$_2$ on a rough surface, and TiO$_2$ pressed on glass. None of the samples showed measurable long distance adhesion forces towards the colloid; therefore, models such as DMT, JKR, and MD, which require adhesion for proper fitting, were excluded (Fig. 5c).

The Hertz model is described by the following equation:

1 $$\begin{aligned} F_{{\mathrm{Hertz}}}=\frac{4}{3}\frac{E^*}{1-\nu ^{*2}}R_{Tip}^{\frac{1}{2}}(s_0-s)^\frac{3}{2}, \end{aligned}$$

where $E^*$ and $\nu ^*$ are the Young’s modulus and Poisson’s ratio of the powder sample, respectively; $R_{Tip}$ is the radius of the glass colloid, $s_0$ is the colloid–sample contact distance, and *s* is the penetration depth. The Hertz model assumes an isotropic and linear elastic solid sample occupying an infinitely extending half space, nondeformable indenter, no additional interactions between the indenter and sample, negligible indentation compared to the sample thickness, absolute elastic behavior, and a homogeneous sample (JPK 2009). Relative to the soft powder films, the indenter is considered nondeformable. Furthermore, the approach curves used for elasticity analysis did not show additional interactions between the colloid and the film.

**Fig. 6:**
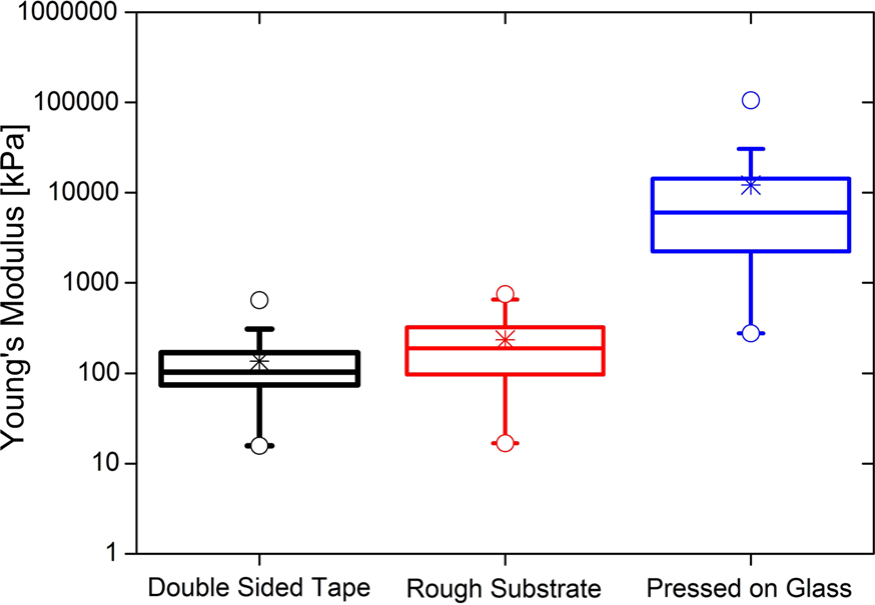
Young’s modulus of TiO$_2$ measured using different sample preparation methods. The double-sided tape and rough substrate preparation methods consist of powder spreading on substrate, and gentle shaking to remove excess powder. The pressed on glass method squeezes the powder between two flat pieces of glass. The *asterisks* are the mean values and the *empty circles* are the maximum and minimum values. The *box* encompasses the second and third quartiles, divided by a *line* corresponding to the median. The *top* and *bottom* whiskers are the outliers with coefficient 1.5

The calculated Young’s modulus for the three sample preparation methods agrees with the hypothesis developed based on the level of powder manipulation (Fig. 6). For the three TiO$_2$ samples, 115, 155, and 258 curves were measured for the pressed on glass, rough substrate, and double-sided tape, respectively. Due to the inhomogeneity of the film, hundreds of measurements were taken to obtain a statistical representation of each sample. The lower values correspond most likely to film spots far from the ideal Hertz assumptions where the measured location had a lower concentration of agglomerates with a nonuniform solid distribution, which explains the wide distribution, while the higher ones are probably closer to the elastic Young’s modulus. The moduli of the double-sided tape and rough substrate are in the same order of magnitude, with a slightly wider distribution for the rough substrate, and the maximum and minimum values similar to those of the double-sided tape. Nonetheless, the pressed on glass sample has a Young’s modulus more than one order of magnitude higher (Fig. 6) as a consequence of the denser film made by pressing. The pressed on glass sample also shows a wider distribution, which could arise from the loose agglomerates present on the surface (Fig. 3b), or any film defect caused by uneven compression or irregular release behavior. Therefore, we have selected the double-sided tape technique as the most reliable sample preparation method.

### Elasticity of different materials

The elasticity of the nanopowder depends on the particle packing, size distribution, shape, surrounding conditions, powder processing, and contact forces. Three common oxide nanopowders of different materials and primary particle sizes were studied, namely Al$_2$O$_3$, SiO$_2$, and TiO$_2$. The packing density depends on the size distribution, powder processing, and particle shape, affecting the space available for particle rearrangement. Additionally, the interparticle forces are affected by material properties such as the Hamaker coefficient and surface hydrophobicity. Thus, each of the three powders has an elasticity which depends on the unique material properties and particle morphology.

**Fig. 7:**
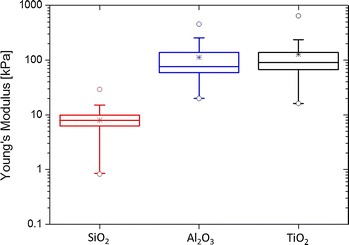
Young’s modulus measured for TiO$_2$, SiO$_2$, Al$_2$O$_3$ on double-sided tape

The Young’s modulus of the powders was calculated by fitting the Hertz model to 219, 305, and 158 curves for SiO$_2$, Al$_2$O$_3$, and TiO$_2$, respectively. Two of the materials, Al$_2$O$_3$ and TiO$_2$, showed a Young’s modulus within the same range in the order of 100 kPa, while SiO$_2$ was an order of magnitude lower (Fig. 7), and with a noticeably narrower distribution. The low Young’s modulus means that the SiO$_2$ agglomerate layer is easier to compress. During the measurements, the force applied on the sample by the colloid is specified; this force is directly proportional to the Hamaker coefficient and elastic deformation of the sample (Tsai et al. 1991). Since the Hamaker coefficient of silica (SiO$_2$) is about an order of magnitude smaller than that of Al$_2$O$_3$ and TiO$_2$ (Bergstrom 1997), a more prominent deformation was expected and indeed obtained, describing a soft, highly elastic material. Furthermore, other factors such as the degree of particle surface roughness and porosity could contribute to the low Young’s modulus of SiO$_2$, and should be further investigated.

As seen from Fig. 7, the values show a wide distribution. This distribution is typical for AFM measurements. Even in the case of perfectly flat substrates such as mica or silicon, AFM values always show wide distribution based on a different number of molecules coming into contact at each measurement (Butt et al. 2005; Farshchi-Tabrizi et al. 2006). However, in the case of porous agglomerates, the contact scenario shows even more variation between measurements since the sample surface is rather heterogeneous, leading to a wider range of measured values.

### Theoretical elasticity

The elasticity of each powder was calculated theoretically using the model of Kendall et al. (1987). According to Kendall’s model, the effective elasticity of the powder sample can be estimated from

2 $$\begin{aligned} E^*=17.1\phi ^4\left[ \frac{E^2\Gamma }{d_p}\right] ^{1/3}, \end{aligned}$$

where $\phi$ is the solid volume fraction, and *E*, $\Gamma$, and $d_p$, the Young’s modulus, work of adhesion, and diameter of the nanoparticles, respectively. This model was developed for anisotropic packing of spherical particles into complex structures with different shapes. All shapes fell into one curve represented by Eq. (2) where the coefficient 17.1 is found from the fit. The dependence of the effective Young’s modulus on the solid fraction to the fourth power arises from a square dependence on the shear modulus (*G*), and a second one on the coordination number ($N_{Co}$).

The applicability of the model to highly porous nanoparticle agglomerates was evaluated. The coordination number of porous structures with solid fraction between 0 and 0.1 still has a square dependence as estimated from the literature (Norman 1971). The original expression to calculate the coordination number is an exponential sum that leads to the Taylor series $N_{Co}=1.99+0.59\phi +11.02\phi ^2-0.02\phi ^3+10.27\phi ^4+O(\phi ^5)$, which results in a parabola for small $\phi$. Additionally, the square influence of the density packing on the modulus described for the material with a random distribution of isolated spherical holes (Mackenzie 1950) could still apply to highly porous structures with randomly distributed particle chains such as nanoparticle agglomerates. A Taylor expansion of the original formula reads as

3 $$G= \sum\limits^{\infty }_{n=1} \frac{4\times 3^{n-1}{k_0^n}{\mu _0}{\phi^n}}{(3k_{0} +4\mu_{0})^n},$$

where $k_0$ is the bulk modulus and $\mu _0$ the shear modulus, which can be taken as a quadratic polynomial for solid fractions in the nanoparticle agglomerate range since terms with higher degrees lead to values more than two orders of magnitude smaller. Therefore, we believe that Kendall’s model can be used to estimate the elasticity of structures with solid fractions lower than 0.1 such as the highly porous nanopowder layers presented in this work.

**Fig. 8:**
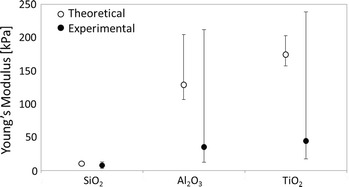
Experimental and theoretical values of the Young’s moduli for SiO$_2$, Al$_2$O$_3$, and TiO$_2$ on double-sided tape. Experimental values obtained from AFM measurement (*solid circles*), and theoretical from Eq. (2) (*empty circles*) are shown. Error bars are the standard deviation of the curves used to calculate the log-normal experimental elasticity, and the standard deviation from the nanoparticle size distribution as estimated from TEM images

For hydrophilic TiO$_2$ (P25), with a solid fraction of 0.03 (Tahmasebpoor et al. 2013), work of adhesion of 0.8 J/m$^2$ (Navrotsky 2003; Kendall et al. 1987), particle diameter of 22 nm (Fig. 1), and particle elasticity of 234 GPa (Chen et al. 2009), we obtain a Young’s modulus of  174 kPa, which is in close agreement with the results from the AFM. The values used for SiO$_2$ (A130) and Al$_2$O$_3$ (AluC) are shown in Table 2, resulting in Young’s moduli of 10 and 129 kPa, respectively. The work of adhesion is calculated as twice the surface energy of the material, which is taken from Navrotsky’s paper (Navrotsky 2003). Since the estimation of surface energy depends on the experimental method and conditions showing strong variations in literature, the paper of Navrotsky et al. was chosen as it includes all three powders used in this study. The theoretical and experimental values are compared in Fig. 8, where the empty circles correspond to the theoretical values with bars representing the spread arising from the nanoparticle size distribution, and the solid circles representing the experimental mode with error bars as the standard deviation for log-normal distribution of the data.

**Table 2:** Values used for the estimation of the effective Young’s modulus

material	*ϕ* ^a^	$\Gamma$ (J/m$^2$]$^{\mathrm{b}}$	*E* (GPa)$^{\mathrm{c}}$	$d_p$ (nm)
TiO$_2$—P25	0.03	0.8	234	22 ± 8
Al$_2$O$_3$—AluC	0.02	5.2	400	8 ± 2
SiO$_2$—A130	0.02	0.18	70	16 ± 6

^a^Solid volume fraction (Tahmasebpoor et al. 2013)

^b^Nanoparticle work of adhesion (Navrotsky 2003)

^c^Nanoparticle Young’s modulus (Chen et al. 2009; Kendall et al. 1987)

Kendall’s model can estimate the elasticity of the highly porous sample to the right order of magnitude, which is known to be extremely challenging. The slight discrepancy between the theoretical and experimental Young’s modulus values can be attributed to the partial plasticity of the agglomerates and the rough distribution of powder throughout the film. In all three cases, the experimental value is lower than the theoretical one since plastic deformation based on aggregate rearrangements during agglomerate compression by the colloid is not accounted for in Kendall’s model. This plasticity of the system must be too small (compared to the spring constant of the cantilever) for the experimental equipment and data analysis software to find the elastic Herzt model unsuited. Furthermore, the spread of the measured values also represents the range of agglomerate properties found throughout the film.

The parameters needed to calculate the elasticity for the different sample preparation methods are presumably known for the rough substrate and double-sided tape, and assumed to be the same; however, the porosity of the pressed on glass sample is unknown. A porosity of $91\pm 5$ % was back-calculated from the Kendall model for TiO$_2$ pressed on glass sample using the AFM measurements as the effective Young’s modulus (Fig. 6). This means that even after the squeezing step, the pressed powder shows a highly porous morphology, which from the SEM images (Fig. 3c) seems appropriately described by the estimated value.

Other theoretical models to compute the Young’s modulus were considered (Adachi et al. 2006; Wagh et al. 1991; Pabst et al. 2006; Kupkova 1993; Yoshimura et al. 2007; Choren et al. 2013). Nevertheless, some of them include fitting parameters that require experimental data (Hasselman 1962; Wang 1984; Martin and Haynes 1971; Phani and Niyogi 1987; Choren et al. 2013), which defeats the purpose of the analytical calculation for this study, and those from Yoshimura et al. (2007) use as parameters properties of the porous material that are still unknown due to technical limitations similar to those encountered for the Young’s modulus (Kovacik 2001). Alternative models, listed in Choren et al.’s review (Choren et al. 2013), which only depend on agglomerate porosity and Young’s modulus of the nonporous material estimate moduli in the gigapascal (GPa) range (see Fig. S4, Supporting Information), more than four orders of magnitude higher than the experimental values obtained from the AFM.

## Conclusions

The research presented in this paper describes a method to experimentally determine the Young’s modulus of structures with porosity higher than 90 %. The focus of the study is on nanoparticle agglomerates, which are a few hundred micrometers in size and very fragile, formed due to strong attractive interactions among the primary particles. The experiments are done by AFM on five different samples including three materials (Al$_2$O$_3$, SiO$_2$, TiO$_2$) using the double-sided tape sample preparation method, and three sample preparation methods (pressed on glass, rough surface, double-sided tape) for one of the nanopowders (TiO$_2$). The results validate the applicability of Kendall et al. model to predict the elasticity of nonspherical highly porous structures. A more detailed analysis on the extrapolation of Kendall’s model to low solid fractions and/or irregularly shaped particles will lead to a better understanding of the solid fraction’s effect on the effective elasticity of porous structures. The proposed experimental technique can be used for validation of current or future models.

## Electronic supplementary material

Below is the link to the electronic supplementary material.

Supplementary material 1 (pdf 1013 KB)
