# Supplementary material for: Contact mechanics of highly porous oxide nanoparticle agglomerates
Source: J Nanopart Res. 2016 Jul 18;18:200. doi: 10.1007/s11051-016-3500-4 (PMC4949302; doi:10.1007/s11051-016-3500-4)
Supplement: Supplementary file 1 — Supplementary material 1 (pdf 1013 KB) [file 11051_2016_3500_MOESM1_ESM.pdf]

Supporting Information

Contact Mechanics of Highly Porous Oxide Nanoparticle Agglomerates

Andrea Fabre[a], Samir Salameh[a], Lucio Colombi Ciacchi[b], Michiel T. Kreutzer[a], J. Ruud van Ommen[a]*

[a] Department of Chemical Engineering, Delft University of Technology, Julianalaan 136, Delft 2628 BL, The Netherlands

[b] Hybrid Materials Interfaces, University of Bremen, Am Fallturm 1, Bremen 28359, Germany

**Corresponding author:* J.R.vanOmmen@tudelft.nl

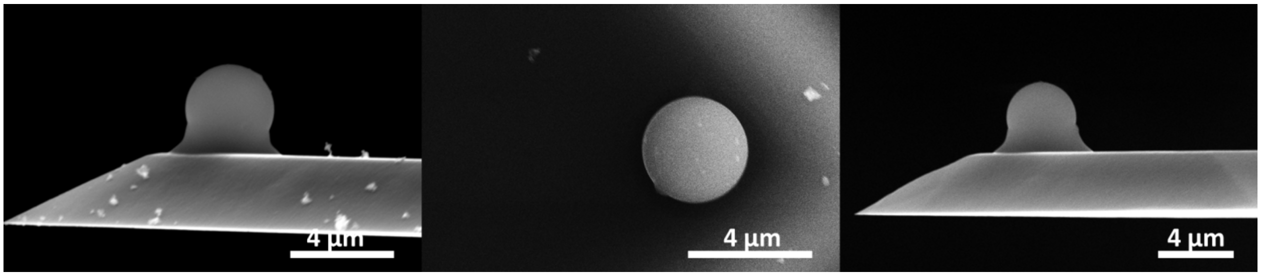

Figure S1. SEM images of used cantilevers, as shown, on rough substrate, double sided tape, and pressed on glass, respectively. The cantilevers were bought from sQube (CP-FM-SiO-B) with a glass colloid of 3.5µm in diameter attached to it.

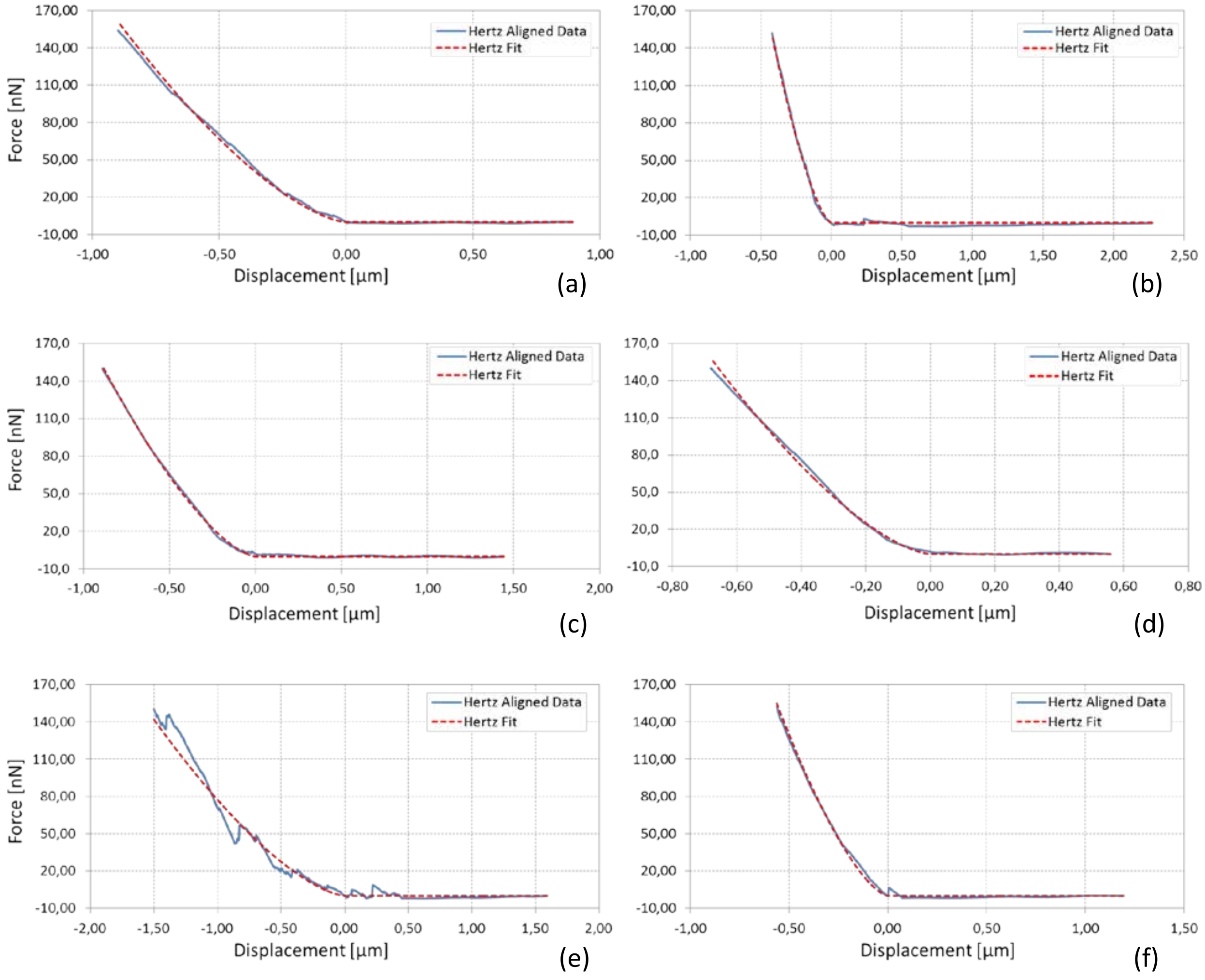

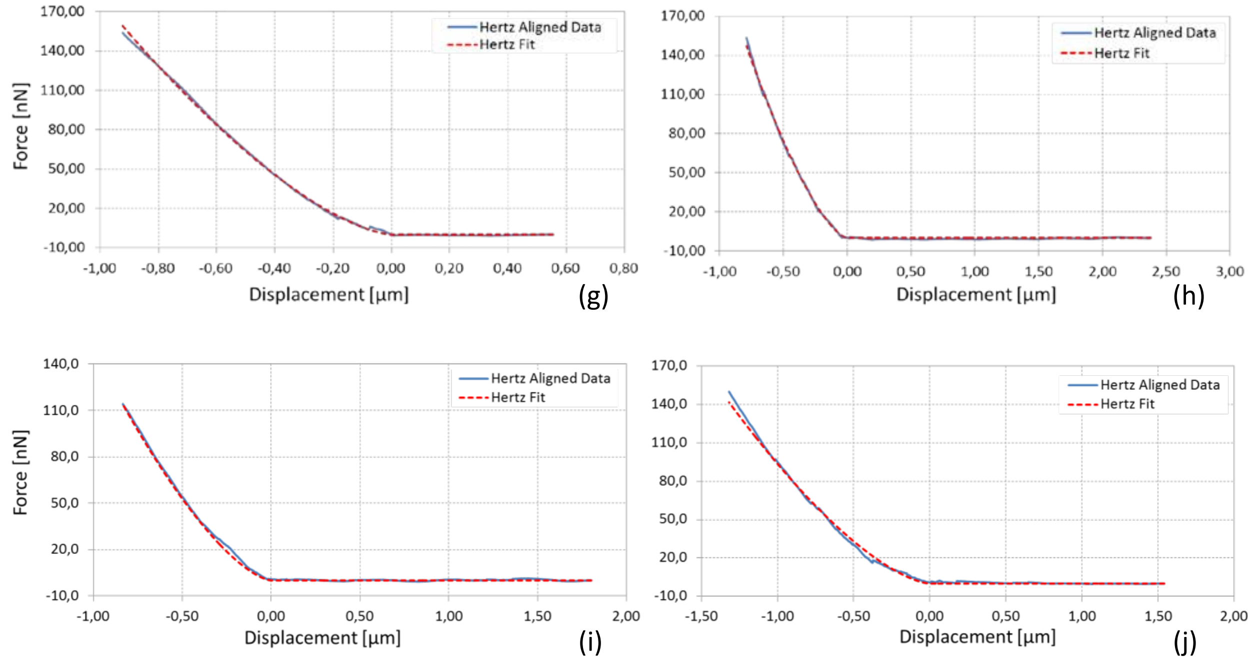

Figure S2. Hertz model fitting of multiple force curves obtained from AFM measurements of titania-TiO_2 (a-d), alumina-Al_2O_3 (e-h), and silica-SiO_2 (i-j) samples on double sided tape using a silica colloid with a diameter of 3.75 µm.

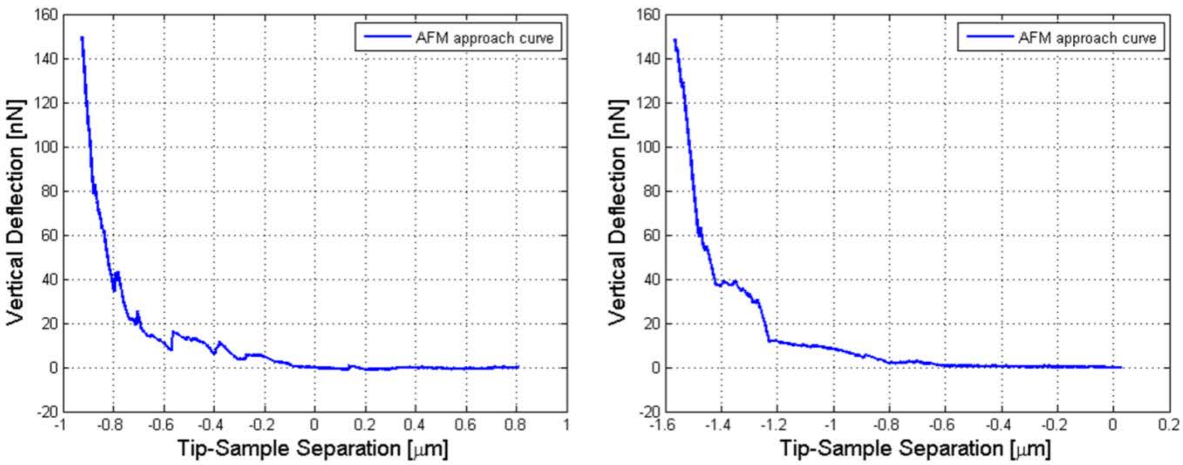

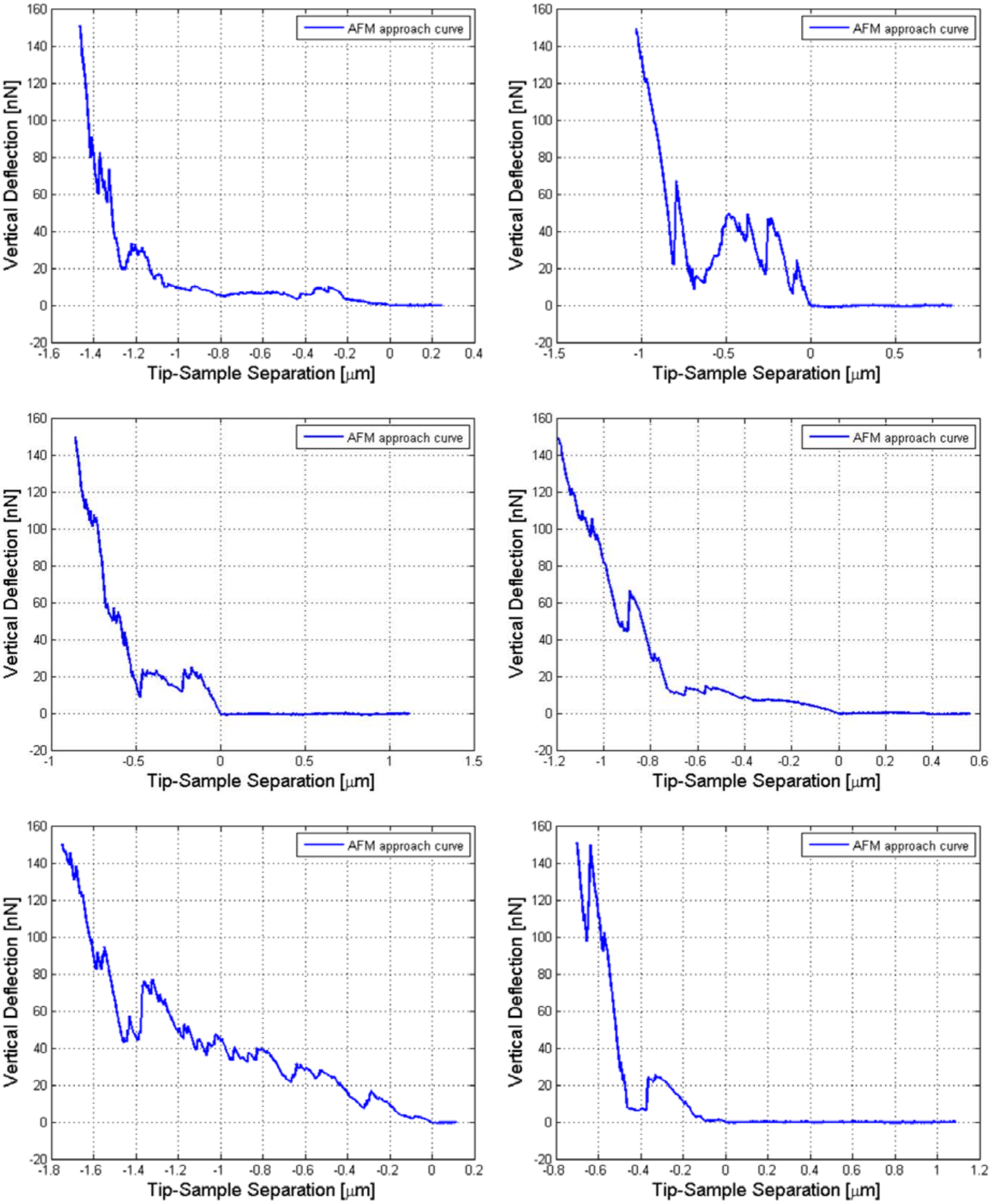
AFM approach curve
Vertical Deflection [nN]
Tip-Sample Separation [μm]

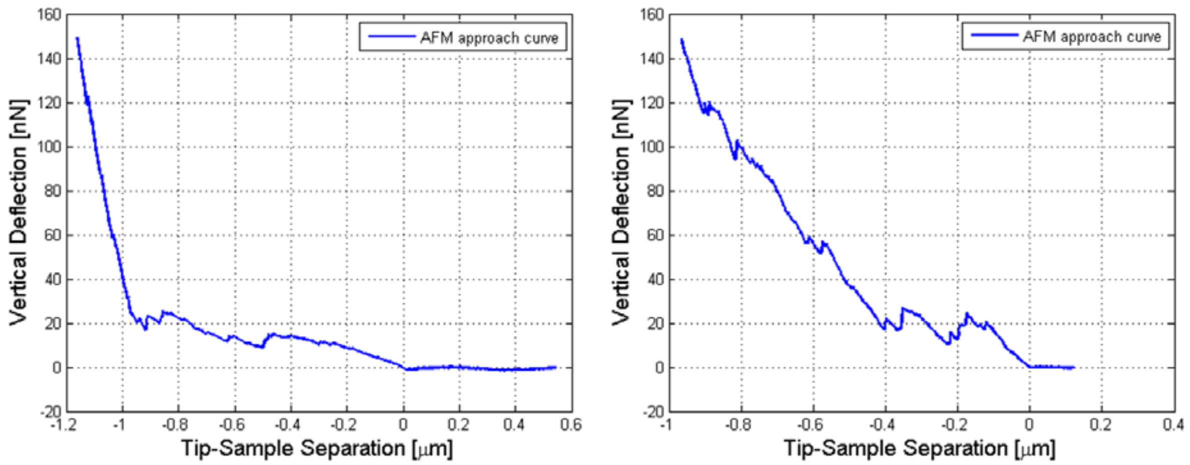

Figure S3. Curves showing behaviors such as particle rearrangement, not suitable for Hertz fitting. Example of curves not considered for measurement, but saved for further analysis of the nanopowder film morphology.

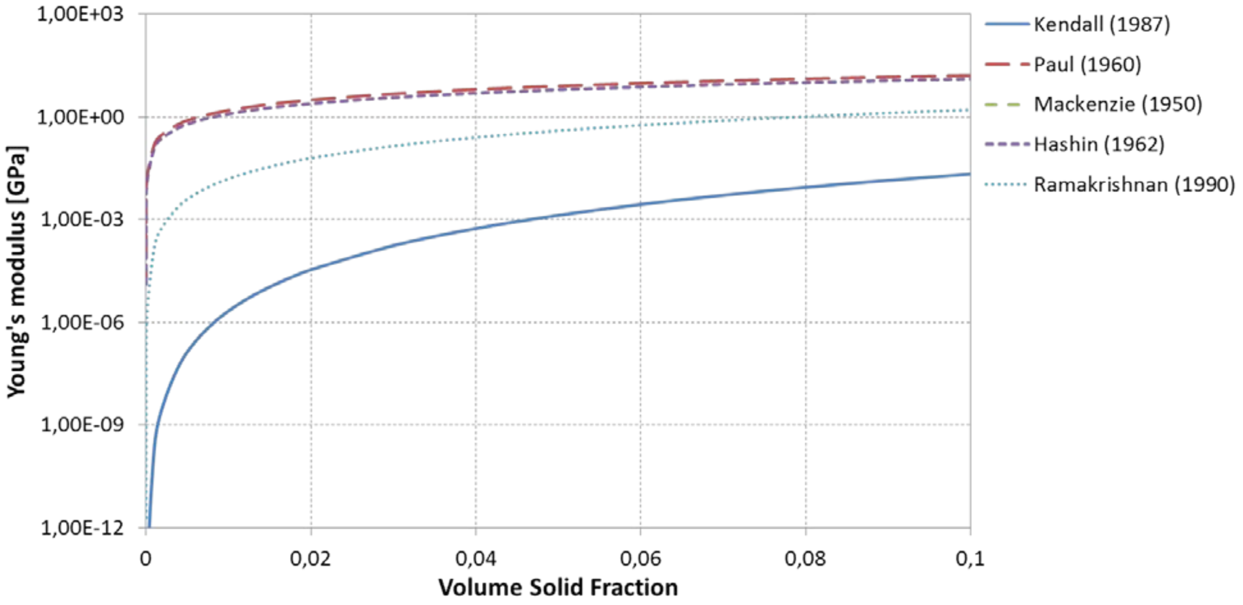

Figure S4. Comparison of models that estimate the elasticity of porous structures without requiring fitting parameters, using porosity as the key parameter. Properties of TiO_2 (P25) were used as parameters for the models.
